# Isolated aortic valve–sparing aortic root replacement in elective patients with bicuspid aortic valve: A single-center experience

**DOI:** 10.1016/j.xjon.2026.101696

**Published:** 2026-02-20

**Authors:** Karl Christian König, Nina Feirer, Miriam Lang, Julia Ehras, Anatol Prinzing, Ulf Herold, Ralf Günzinger, Rüdiger Lange, Markus Krane, Keti Vitanova

**Affiliations:** a Department of Cardiovascular Surgery, Institute Insure, German Heart Center Munich, School of Medicine & Health, Technical University of Munich, Munich, Germany; b Department of Cardiovascular Surgery, University Medicine Frankfurt, Goethe University Frankfurt, Frankfurt, Germany; c Department of Thoracic and Cardiovascular Surgery, West German Heart and Vascular Center, University Medicine Essen, Essen, Germany; d DZHK (German Centre for Cardiovascular Research), Partner Site Munich Heart Alliance, Munich, Germany

**Keywords:** aortic surgery, aortic valve, valve-sparing procedure

## Abstract

**Objective:**

The David-I procedure is the standard surgical treatment for patients with aortic root aneurysms and preserved aortic valve morphology. Its application in patients with bicuspid aortic valves presents additional challenges. This study compares long-term outcomes of the elective, isolated David-I procedure in patients with bicuspid aortic valves and tricuspid aortic valves.

**Methods:**

We retrospectively reviewed all elective patients undergoing an isolated David-I procedure between 2004 and 2019. Patients with bicuspid aortic valves or tricuspid aortic valves and isolated aortic root aneurysms with or without aortic regurgitation due to annular dilatation were included, independent of severity. Patients with structural aortic valve disease were scheduled for a Bentall procedure. Surgical indications followed European Society of Cardiology and American College of Cardiology/American Heart Association guidelines. Emergency and concomitant procedures were excluded. Follow-up data were obtained from patient questionnaires and echocardiographic reports from referring cardiologists.

**Results:**

Thirty-five patients with bicuspid aortic valves and 134 patients with tricuspid aortic valves were included. Patients with bicuspid aortic valves were significantly younger (45.3 ± 11 vs 51.8 ± 12 years, *P* = .002). Median follow-up was 10 years (interquartile range, 4-12). Mortality was 0% in the bicuspid aortic valve group and 6.7% in the tricuspid aortic valve group (*P* = .002). Redo procedures were required more frequently in the bicuspid aortic valve group (22.9% vs 7.5%, *P* = .014). Moderate to severe aortic regurgitation occurred in 25.7% of the bicuspid aortic valve group and 18.7% of the tricuspid aortic valve group, without significant difference.

**Conclusions:**

The elective, isolated David-I procedure can be performed with excellent long-term survival in patients with bicuspid aortic valves. However, compared with tricuspid valves, patients with bicuspid aortic valves show a higher rate of redo procedures, indicating the need for careful patient selection and long-term follow-up.

## Graphical Abstract

Early results: Reduction of AR in patients with BAVs immediately after the David-I procedure. Follow-up results: Kaplan–Meier analysis showing a significantly lower freedom from reoperation in patients with BAVs over 10 to 15 years after the David-I procedure (log-rank *P* = .002). Created with Biorender.com.

**Figure:**
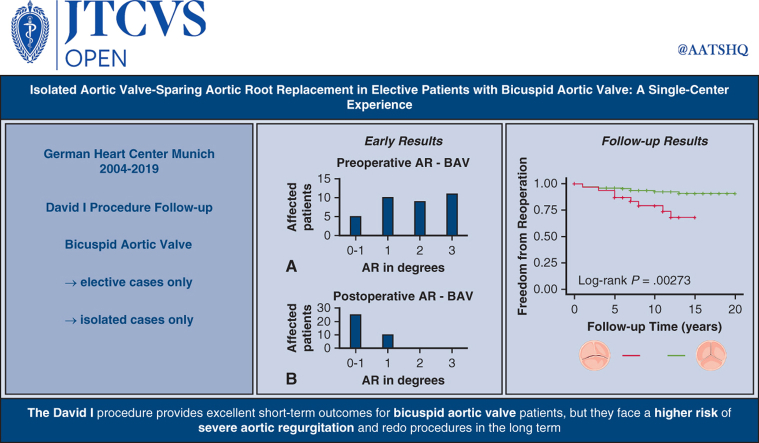

**Figure:**
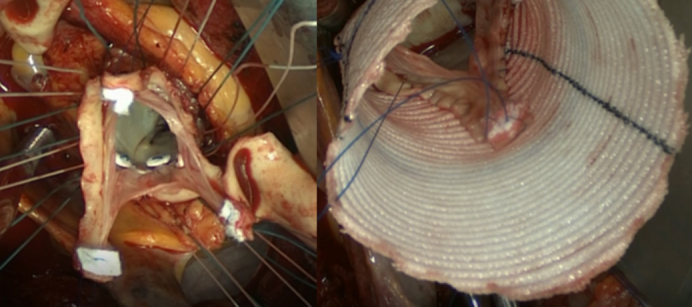
David-I: *Left*: preserved aortic valve, resected sinuses. *Right*: implanted tube graft.

**Central Message**

The David-I procedure provides excellent short-term outcomes for patients with BAVs, but they face a higher risk of severe AR and redo procedures in the long term.

**Perspective**

The elective David-I is safe and effective in patients with BAVs, with low perioperative risk and good survival. Yet, patients with BAVs, especially with severe preoperative regurgitation, face a higher risk of late valve failure and more reinterventions. Careful selection and a low threshold for Bentall conversion are key to optimize outcomes.

Valve-sparing aortic root replacement (VSARR), introduced in 1992 by Drs David and Feindel, was initially applied solely to patients with morphologically normal aortic valves.^1^ This technique showed excellent short- and long-term results in elective patients with tricuspid aortic valves (TAVs).^2^^,^^3^ Although it has been extensively used in the meantime, the indication for this procedure has been extended gradually to patients with aortic dissections, moderate-to-severe aortic regurgitation (AR), and bicuspid aortic valves (BAVs).^4^^,^^5^ Data on long-term outcomes after VSARR procedures, in terms of long-term performance and durability in patients with BAV, remain conflicting and are still a matter of discussion.^6^, ^7^, ^8^, ^9^, ^10^, ^11^, ^12^ The purpose of this retrospective study was to analyze the outcomes, with particular emphasis on long-term results, in patients with BAVs who underwent VSARR. These results were compared with those of patients with TAVs who also underwent VSARR. In contrast to many previously published studies on this topic, we applied a different methodological approach by exclusively analyzing elective patients who underwent an isolated procedure. The end points assessed were the need for reoperation due to aortic valve degeneration and all-cause mortality. By doing so, we deliberately accepted smaller patient numbers in favor of potentially less biased data.

## Material and Methods

### Patients

We retrospectively reviewed the data of patients who underwent the David-I procedure between 2004 and 2019 at our institution. Patients were categorized into 2 groups based on their aortic valve morphology: BAV and TAV. Inclusion criteria included isolated, elective cases that received the procedure. The surgical indication was an aortic root aneurysm, with or without concomitant aortic valve regurgitation, according to European Society of Cardiology and American College of Cardiology/American Heart Association guidelines.^13^^,^^14^ Patients with AR due to dilation of the aortic annulus were considered for a VSARR procedure, regardless of the severity of the AR. Patients with aortic stenosis or AR resulting from structural valve degeneration were excluded from the VSARR procedure. Additional exclusion criteria included concomitant or emergency procedures. Data from both groups were subsequently compared. Four experienced senior surgeons performed all procedures. In this observational cohort study, data were gathered retrospectively, including follow-up information.

### Surgical Technique

All patients underwent median sternotomy for optimal visualization. After the patients were placed on cardiopulmonary bypass, cold crystalloid cardioplegic solution was administered. The aneurysmatically altered aorta was opened longitudinally. The diseased portion of the ascending aorta and the aortic root were resected. The aortic root was gently mobilized down to approximately 2 mm below the aortic annulus. The coronary ostia were preserved as buttons for later reimplantation into the aortic graft. The aortic sinuses were resected to a margin of 5 mm above the aortic annulus. In case of aortic insufficiency due to a prolapsing cusp, aortic valve repair was performed by plicating the corresponding cusp at the commissure level to the adjacent healthy cusp. The aortic graft size was measured using specific aortic tube graft sizers.

Subsequently, 12 to 16 pledgeted sutures of 2-0 polyester fiber (Ethibond, Ethicon Inc) were placed circumferentially in an inside-out manner below the aortic valve. By tying the aforementioned sutures, the tube graft was fixed below the aortic annulus. The aortic sinuses were fixed to the graft using 3 double-armed 4-0 propylene sutures (Prolene, Ethicon Inc) in a continuous or running manner. The coronary ostia were then reimplanted with 5-0 propylene sutures (Prolene). Finally, distal graft anastomosis was performed with a 4-0 running suture (Prolene). All patients received intraoperative transesophageal echocardiography to assess the procedural result.

### Follow-up

Postoperative aortic valve findings were obtained through transthoracic echocardiography. Echocardiographic follow-up data were gathered from the respective cardiologists. Follow-up data were collected according to established guidelines.^15^ We categorized the AR as nonexistent or trivial, mild, moderate, or severe following common echocardiographic guidelines.^16^^,^^17^ Borderline findings were assigned to the next higher category. Therefore, any presence of aortic stenosis or moderate or higher AR was considered as structural valve deterioration. A questionnaire was used to interview the patients about their current health status based on the New York Heart Association (NYHA) classification. The questionnaire also assessed any major cardiovascular adverse event (MACE), as well as possible redo procedures or additional cardiac surgeries. MACE was defined as a 2-point event, including nonfatal stroke and nonfatal myocardial infarction, excluding cardiovascular death. Mortality data are reported exclusively as all-cause mortality because of incomplete data on cardiovascular-specific death rates.

### Statistics

All statistical analyses were performed using IBM SPSS 25. Normal distribution of continuous data was tested using the Shapiro-Wilks test. Normally distributed data are presented as mean with corresponding SD, and nonparametric data are presented as median with interquartile range (IQR) (25th-75th percentile). Analysis of continuous data from the 2 groups was performed using the *t* test or Mann–Whitney *U* test, as appropriate. Freedom from reoperation and survival analyses were performed using Kaplan–Meier analysis. The log-rank test was used to evaluate differences.

### Ethics Approval

Data gathering was approved by the local ethics committee of the School of Medicine and Health at the Technical University of Munich (Project nos. 2025-390-S-CB, date: July 23, 2025). All study procedures conformed to the ethical standards of the Declaration of Helsinki. Because of the retrospective design and the use of routinely collected clinical data, the requirement for informed consent was waived by the ethics committee.

## Results

### Baseline Demographics and Perioperative Outcome

Between 2004 and 2019, 35 patients with BAV and 134 patients with TAV morphology received an elective, isolated aortic valve-sparing aortic root replacement (VSARR, David-I procedure) with a straight tube graft at our department. Patient characteristics are shown in Table 1. We could gather 100% follow-up data of the patients with BAV and TAV. In this context, we also gathered 100% of the echocardiographic follow-up results from the referring cardiologists. We analyzed only the latest echocardiography results as part of the follow-up data-collection process. Median follow-up time was 10 years in both groups (TAV 10 years,^6^, ^7^, ^8^, ^9^, ^10^, ^11^, ^12^ BAV 10 years^4^, ^5^, ^6^, ^7^, ^8^, ^9^, ^10^, ^11^, ^12^).

**Table 1:** Patient characteristics: Analysis of metric data was done using the Mann–Whitney *U* test and analysis of categoric data was done using the chi-square analysis

Characteristics	TAV (n = 134)	BAV (n = 35)	*P* value
Age (y), mean ± SD	51.8 ± 12	45.3 ± 11	.002
Male sex, n (%)	107 (79.9)	32 (91.4)	.110
Height (cm), mean ± SD	178 ± 9.5	180 ± 8.7	.080
Weight (kg), mean ± SD	88.8 ± 16.7	83.1 ± 11.6	.035
Body surface area (m^2^), mean ± SD	2.06 ± 0.22	2.03 ± 0.17	.441
Body mass index (kg/m^2^), mean ± SD	28 ± 4.4	26 ± 3.4	.003
Previous cardiac procedure	0	0	
Cardiovascular risk factors			
Arterial hypertension, n (%)	83 (61.9)	16 (45.7)	.080
Hyperlipoproteinemia, n (%)	37 (27.6)	6 (17.1)	.200
Diabetes mellitus type 2, n (%)	5 (3.7)	2 (5.7)	.600
Obesity (BMI ≥30 kg/m^2^), (%)	40 (29.9)	3 (8.6)	.010
Nicotine, n (%)	50 (37.3)	9 (25.7)	.190
NYHA classification			
NYHA I, n (%)	23 (17.2)	13 (37.1)	.010
NYHA II, n (%)	67 (50)	16 (45.7)	.650
NYHA III, n (%)	44 (32.8)	6 (17.1)	.070
NYHA IV, n (%)	0	0	
Secondary diagnoses			
Chronic obstructive pulmonary disease	1 (0.9)	0	
Coronary artery disease, n (%)	1 (0.9)	1 (2.8)	.300
Carotid artery disease, n (%)	2 (1.8)	0	
Peripheral artery disease, n (%)	0	0	
Chronic kidney disease, n (%)	3 (2.6)	0	
Status after stroke, n (%)	3 (2.6)	1 (2.8)	.830
Status after myocardial infarction, n (%)	0	0	
Degenerative tissue disorder, n (%)	7 (5.2)	0	
Marfan syndrome, n (%)	7 (5.2)	0	
Echocardiography			
Left ventricular EF in %, mean ± SD	58 ± 8.8	61 ± 12	.054
EF classification			
Normal (50-70%), n (%)	124 (92.5)	33 (94.2)	.719
Mild reduction (40-49%), n (%)	6 (4.5)	0	
Moderate reduction (30-39%), n (%)	4 (3.0)	1 (2.8)	.973
Severe reduction (<30%), n (%)	0	1 (2.8)	
Preoperative aortic valve function			
No or trivial aortic regurgitation, n (%)	7 (5.2)	5 (14.2)	.060
Mild aortic regurgitation, n (%)	26 (19.4)	10 (28.4)	.238
Moderate aortic regurgitation, n (%)	48 (35.8)	9 (25.7)	.260
Severe aortic regurgitation, n (%)	53 (39.6)	11 (31.4)	.377
Aortic stenosis, n (%)	0	0	
Indication for surgery			
Aortic aneurysm (mm), mean ± SD	57.2 ± 7.9	55 ± 8.8	.030
Aortic dissection	0	0	

*TAV*, Tricuspid aortic valve; *BAV*, bicuspid aortic valve; *BMI*, body mass index; *NYHA*, New York Heart Association; *EF*, ejection fraction.

The mean age of the patients with BAVs was 44.5 ± 11 years, and 91% were male. In the TAV cohort, the mean age was 51.8 ± 12 years, and 79.9% were male (*P* = .002). None of the patients with BAV had a degenerative tissue disorder such as Marfan syndrome, whereas 7 patients in the TAV cohort were diagnosed with Marfan syndrome. The mean size of the aortic aneurysm was 55.1 ± 8.8 mm in the BAV group and 57.2 ± 7.9 mm in the TAV group. Approximately one-third of the patients presented with mild, moderate, or severe AR (10, 9, and 11 patients, respectively). Preoperative AR rates are presented in Table 1.

Intraoperative data are presented in Table 2. The mean cardiopulmonary bypass time was 167.3 ± 31.1 minutes, and the mean aortic crossclamp time was 132.6 ± 23.2 minutes in the BAV group. No statistically significant differences in cardiopulmonary bypass or aortic crossclamp times were found between the TAV and BAV groups (Table 2).

**Table 2:** Intraoperative data: Analysis of metric data was done using the Mann–Whitney *U* test and analysis of categoric data was done using the chi-square analysis

Characteristics	TAV (n = 134)	BAV (n = 35)	*P* value
Cardiopulmonary bypass time (min), median [IQR]	160.5 [142-185]	168 [150-182]	.284
Aortic crossclamp time (min), median [IQR]	129 [113-145]	133.5 [114-147]	.241
Sievers classification of the aortic valve, n (%)			
Type 0, n (%)		3 (8.5)	
Type 1, n (%)		32 (91.4)	
Fusion of right and left coronary cusp, n (% type I)		20 (57.1)	
Fusion of non and right coronary cusp, n (% type I)		8 (22.8)	
No data available on cusp fusion, n (% type I)		4 (11.4)	
Type 2, n (%)		0	
Prosthesis size			
Mean prosthesis size (mm), mean ± SD	31 ± 1.9	31 ± 1.9	.188
26, n (%)	1 (0.7)	0	
28, n (%)	17 (12.6)	3 (8.5)	.502
30, n (%)	37 (27.6)	12 (34.2)	.438
32, n (%)	55 (41.0)	11 (31.4)	.299
34, n (%)	20 (14.9)	9 (25.7)	.131
Additional procedures			
Additional aortic valve plasty, n (%)	30 (22.4)	20 (57.1)	<.001
Concomitant cardiac procedures, n (%)	0	0	

*TAV*, Tricuspid aortic valve; *BAV*, bicuspid aortic valve; *IQR*, interquartile range.

Aortic valve morphology was classified according to the Sievers classification.^18^ Three patients were classified as Sievers type 0, and 32 patients were classified as Sievers type 1, with the majority showing fusion of the right and left coronary cusps (20 patients). None of the patients exhibited Sievers type 2 morphology. The implanted straight tube prostheses had identical mean sizes of 31 ± 1.9 mm in both groups.

In the BAV group, 20 patients (57.1%) underwent additional aortic valve cusp repair compared with 30 patients (22.4%) in the TAV group (*P* < .001). The median intensive care unit stay was 2 days (IQR, 1-4), and the mean hospital stay was 11.2 ± 4.8 days. These results showed no statistically significant differences compared with the TAV cohort (*P* = .301 and .248, respectively).

Two patients required reexploration for significant postoperative bleeding. Temporary neurologic deficits occurred in 2 patients, but no patient experienced permanent neurological deficits. The perioperative and 30-day mortality rates were 0% in the BAV cohort. By contrast, 1 patient in the TAV group died during the hospital stay.

**Figure 1:**
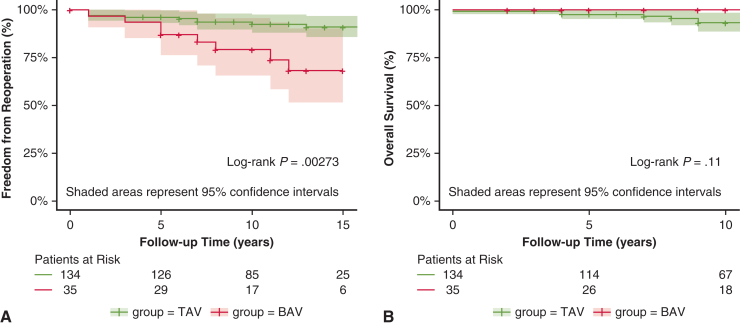
A, Kaplan–Meier analysis for freedom from valve-related reoperation of patients undergoing David-I procedures with BAV and TAV. Log rank: *P* = .002. *Red line* shows patients with BAVs, and *blue line* shows patients with TAVs. B, Kaplan–Meier survival analysis of patients undergoing David-I procedures with BAVs and TAVs. Log rank: *P* = .11. *Red line* indicates patients with BAVs, and *blue line* indicates patients with TAVs. *BAV*, Bicuspid aortic valve; *TAV*, tricuspid aortic valve.

Echocardiographic examinations at discharge revealed a mean left ventricular ejection fraction of 52.8% ± 10.3% compared with a mean ejection fraction of 62% ± 11% on admission. Evaluations of the aortic valve showed that 26 patients had no or trivial AR, and 9 patients had mild AR. Postoperative AR rates determined by echocardiography are displayed in Figure 1, *B*. In summary, there were no statistically significant differences in perioperative outcomes between the BAV and TAV cohorts. Postoperative data are presented in Table 3.

**Table 3:** Postoperative data: Analysis of metric data was done using the Mann–Whitney *U* test and analysis of categoric data was done using the chi-square analysis

Characteristics	TAV (n = 134)	BAV (n = 35)	*P* value
ICU stay (d), median [IQR]	2 [1-4]	2 [1-3]	.301
ICU stay >3 d, n (%)	34 (25.4)	7 (20)	.509
Hospital stay (d), median [IQR]	11 [9-13]	10 [8.5-13]	.248
Mechanical ventilation (h), median [IQR]	8 [6-14]	7 [5-12.5]	.153
Ventilation >3 d, n (%)	3 (2.2)	0	
Postoperative complications			
Rethoracotomy for bleeding, n (%)	11 (8.2)	2 (5.6)	.621
Tracheostomy, n (%)	2 (1.5)	0	
Dialysis, n (%)	1 (0.7)	0	
Temporary neurologic deficit, n (%)	3 (2.2)	2 (5.6)	.279
Permanent neurologic deficit (stroke), n (%)	1 (0.7)	1 (2.8)	.303
Permanent pacemaker implantation, n (%)	3 (2.2)	1 (2.8)	.830
Perioperative death			
All-cause mortality, n (%)	1 (0.7)	0	
Echocardiography			
Left ventricular ejection fraction (EF in %), median [IQR]	55 [50-60]	57.5 [46-60]	.480
EF classification			
Normal (50-70%), n (%)	107 (79.9)	27 (77.2)	.954
Mild reduction (40-49%), n (%)	16 (11.9)	4 (11.4)	.933
Moderate reduction (30-39%), n (%)	7 (5.2)	4 (11.4)	.185
Severe reduction (<30%), n (%)	4 (3)	0	
Postoperative aortic valve function			
No or trivial aortic regurgitation, n (%)	93 (69.4)	25 (71.4)	.816
Mild aortic regurgitation, n (%)	34 (25.4)	10 (28.6)	.701
Moderate aortic regurgitation, n (%)	7 (5.2)	0	
Severe aortic regurgitation, n (%)	0	0	
Maximum gradient (mm Hg)	14.7 ± 3.5	21 ± 6	.040
Mean gradient (mm Hg)	8.9 ± 2.6	10 ± 3	.223

*TAV*, Tricuspid aortic valve; *BAV*, bicuspid aortic valve; *IQR*, interquartile range; *EF*, ejection fraction.

### Long-Term Outcome

Follow-up data from 35 patients were collected and analyzed, achieving a total of 100% follow-up rate. These data are presented in Table 4. The median follow-up time was 10 (IQR, 4-12) years in the BAV group and 10 (6-12) years in the TAV group (*P* = .08). None of the BAV group died during the observational period, and 8 patients (6%) in the TAV group died during follow-up (>90 postoperative days). Kaplan–Meier survival analysis of patients with BAV and TAV who underwent David-I procedures revealed a statistically insignificant lower risk of death for BAV during follow-up, with a *P* value of .11 (log-rank analysis). Kaplan–Meier analyses are shown in Figure 1, *A* and *B*.

**Table 4:** Follow-up data: Analysis of metric data was done using the Mann–Whitney *U* test and analysis of categoric data was performed using chi-square analysis

Characteristics	TAV (n = 134)	BAV (n = 35)	*P* value
Follow-up time (y), median [IQR]	10 [6-12]	10 [4-12]	.08
MACE, n (%)	6 (4.5)	0	
Death during follow-up, n (%)	8 (6)	0	
All-cause mortality, n (%)	9 (6.7)	0	
NYHA classification			
NYHA I, n (%)	81 (60.4)	21 (60)	.961
NYHA II, n (%)	38 (28.4)	10 (28.5)	.980
NYHA III, n (%)	14 (10.5)	4 (11.5)	.866
NYHA IV, n (%)	1 (0.75)	0	
Aortic valve–related redo procedure			
Total redo procedures, n (%)	10 (7.5)	8 (22.9)	.014
Aortic regurgitation, n (%)	7 (5.2)	6 (17.1)	.029
Aortic stenosis, n (%)	1 (0.75)	2 (5.7)	.109
Combined aortic valve pathology	0	0	
Endocarditis, n (%)	2 (1.5)	0	
Type of redo procedure			
Mechanical aortic valve replacement, n (%)	1 (0.75)	2 (5.7)	.109
Biological aortic valve replacement, n (%)	7 (5.2)	5 (14.3)	.13
TAVR, n (%)	2 (1.5)	1 (2.9)	.503
Echocardiography			
Left ventricular ejection fraction (%), median [IQR]	60 [50-65]	NA	
EF classification			
Normal (50-70%), n (%)	115 (86)	27 (77.2)	.212
Mild reduction (40-49%), n (%)	19 (14)	4 (11.4)	.672
Moderate reduction (30-39%), n (%)	0	2 (5.7)	
Severe reduction (<30%), n (%)	0	2 (5.7)	
Postoperative aortic valve function			
Aortic regurgitation, n (%)	45 (33.6)	11 (31.4)	.809
No or trivial aortic regurgitation, n (%)	85 (63.4)	24 (68.6)	.692
I°, Mild aortic regurgitation, n (%)	20 (14.9)	2 (5.7)	.256
II°, Moderate aortic regurgitation, n (%)	18 (13.4)	3 (8.6)	.572
III°, Severe aortic regurgitation, n (%)	7 (5.3)	6 (17.1)	.029
Aortic stenosis, n (%)	4 (3.0)	8 (23.1)	<.001
I°, Mild aortic stenosis, n (%)	2 (1.5)	3 (8.6)	.06
II°, Moderate aortic stenosis, n (%)	1 (0.75)	3 (8.6)	.028
III°, Severe aortic stenosis, n (%)	1 (0.75)	2 (5.7)	.109
Maximum gradient (mm Hg)	17.6 ± 6.3	24 ± 11.3	.086
Mean gradient (mm Hg)	12.9 ± 9.5	21.4 ± 9	.121
Combined aortic valve pathology	0	2	

*TAV*, Tricuspid aortic valve; *BAV*, bicuspid aortic valve; *IQR*, interquartile range; *MACE*, major adverse cardiovascular event; *NYHA*, New York Heart Association; *NA*, not available; *EF*, ejection fraction.

Follow-up echocardiographic evaluations revealed that 24 patients (68.6%) in the BAV group and 85 patients (63.4%) in the TAV group had no or trivial aortic valve regurgitation. In the BAV group, 2 patients had mild AR, 3 patients had moderate AR, and 6 patients had severe AR. Consequently, 25 patients (18.7%) in the TAV group and 9 patients (25.7%) in the BAV group developed moderate-to-severe AR during the follow-up period (*P* = .351). Five patients (14.2%) developed mild aortic stenosis, with a mean maximum pressure gradient of 25 ± 10 mm Hg.

During the follow-up period, 8 patients (22.9%) in the BAV group required aortic valve–related reinterventions compared with 10 patients (7.5%) in the TAV group (*P* = .014). In all BAV cases, the indication for reintervention was significant AR. During follow-up, 8 patients in the BAV group developed aortic stenosis. Of these, 3 (8.6%) had mild, 3 (8.6%) had moderate, and 2 (5.7%) had severe aortic stenosis. Among the 8 patients with BAV requiring redo procedures, 6 (75%) had severe AR and 2 (5.7%) had severe aortic stenosis. Of these, 5 (62.5%) underwent prior concomitant aortic valve plasty. Overall, 77.1% of the patients with BAV did not require any reintervention during the follow-up period. Kaplan–Meier analysis for freedom from valve-related reoperation revealed a higher risk for the BAV group compared with the TAV group, with a *P* value of .002.

None of the BAV group experienced MACE, such as myocardial infarction or stroke, during follow-up. In contrast, 6 patients in the TAV group (4.5%) experienced MACE, including 1 patient who developed Stanford B aortic dissection and 5 patients who had ischemic strokes with permanent neurological deficits. At the follow-up interview, 21 patients (60%) with BAVs were classified as NYHA class I, 10 patients (28.5%) as NYHA class II, and 4 patients as NYHA class III. Thus, 88.5% of the BAV group were classified as NYHA class I or II, similar to 88.8% in the TAV group. Left ventricular ejection fraction remained normal in 77.2% of the BAV group and 86% of the TAV group (*P* = .212).

## Discussion

For the most accurate assessment and comparison of patients undergoing David-I procedures with TAV or BAV, we excluded patients who received emergency or concomitant procedures. We first evaluated the early outcome after VSARR to assess the safety of the procedure in patients with BAVs. Next, we analyzed the long-term outcome. We examined procedure-related complications and long-term mortality rates. Subsequently, we emphasized native aortic valve function and aortic valve–related redo procedures, because BAVs are particularly prone to developing structural valve degeneration.

### Early Outcome

Patients with BAV were significantly younger compared with patients with TAV who underwent VSARR in our study. This is explained by the structural anomaly of BAV, which predisposes patients to develop aortic valve diseases such as regurgitation or stenosis considerably earlier than in patients with TAV.^19^^,^^20^ In light of the young mean age of the patients with BAV, it is not surprising that this cohort was relatively healthy, with 94% having a normal left ventricular ejection fraction and 82% classified as NYHA class I or II. Accordingly, perioperative mortality was excellent, with no patients dying during the hospital stay or within the first 30 postoperative days. As demonstrated in prior studies, the application of the David-I procedure in this specific patient cohort is extremely safe.^10^^,^^21^, ^22^, ^23^ Intraoperatively, 32 patients (91.4%) had Sievers type I BAV, which is consistent with the normal distribution of BAV.^18^ The rate of reexploration for bleeding was lower in the BAV group than in the TAV group (5.6% vs 8.2%), although without statistical significance (*P* = .621). Published data on reexploration in patients with BAV varies significantly, ranging from 1.4 to 14%.^10^^,^^21^, ^22^, ^23^ The postoperative need for a permanent pacemaker was very low, with only 1 patient (2.8%) receiving one, a result comparable to the TAV cohort (2.2%, *P* = .830). Beyond this, no other severe complications after the David-I procedure were observed, such as stroke, acute kidney failure requiring dialysis, or respiratory insufficiency with prolonged ventilation times. Thus, consistent with other clinical trials, we demonstrated excellent short-term results in patients with BAVs undergoing the David-I procedure.^10^^,^^21^, ^22^, ^23^, ^24^

### Long-Term Outcome

Beyond the safety of the David-I procedure, the most critical question concerns the long-term durability of the BAV after this procedure. Thus, we analyzed follow-up data of our BAV patients who underwent David-I procedures. Unlike the TAV cohort, the BAV patients experienced no MACE and no deaths during the follow-up period. Possible explanations for these findings include the significantly younger mean age of the BAV cohort compared with the TAV group and the relatively small sample size of the BAV group.

Cardiovascular risk factors were comparable in both groups. Regarding secondary conditions, slightly more patients in the TAV group were affected. In terms of cardiovascular symptoms, NYHA classification, and left ventricular ejection fraction, both groups showed similar results. Most patients in both groups had a normal ejection fraction and were classified as NYHA I.

However, echocardiographic follow-up evaluations of the aortic valve revealed significantly more patients in the BAV group with severe aortic insufficiency (17.1% vs 5.3% in the TAV group). Consequently, significantly more patients in the BAV group underwent aortic valve–related redo procedures. We present follow-up echocardiographic data for all 35 patients with BAVs, including data from patients who underwent a redo procedure. We are convinced that this approach provides a more realistic representation of long-term aortic valve degeneration. In contrast, other studies have only reported echocardiographic data for event-free patients, which should be considered for accurate comparisons.^2^^,^^22^ None of our event-free patients experienced severe AR, whereas 3 patients (8.6%) in this group had moderate AR. Consequently, our results are comparable to those of other study groups, particularly for patients with AR of I° or less.^2^^,^^22^

Kaplan–Meier analysis for freedom from reoperation also demonstrated a statistically significant difference between the groups, with a log-rank value of 0.002. The rate of freedom from reoperation remained high in the TAV group, at 84% after 15 years, whereas it decreased to 64% in the BAV group after only 10 years. Therefore, our reported rate is considerably lower than in other studies, as well as in a recent meta-analysis by Wilson-Smith and colleagues.^10^^,^^21^^,^^23^^,^^24^ These reported rates of freedom from reoperation at 5 years ranged between 88 and 97%.^10^^,^^21^^,^^23^^,^^24^ However, Beckmann and colleagues^22^ reported a comparable percentage of patients with BAVs who underwent VSARR requiring an aortic valve–related redo procedure during follow-up, with a rate of 20%.

As noted earlier, follow-up results from various studies show significant differences in aortic valve–related redo procedure rates.^10^^,^^21^, ^22^, ^23^, ^24^ Drawing accurate conclusions remains challenging, because many potential influencing factors are not consistently reported. These may include variations in BAV morphology, the proportion of BAV patients undergoing VSARR versus Bentall procedures, Bentall conversion rates, differences in aortic root geometry and size, and the surgical team's expertise in both operative skills and preoperative assessment. According to our findings, we conclude that patients with BAV and severe preoperative AR requiring concomitant aortic valve plasty are particularly prone to develop postoperative severe AR. Therefore, we recommend aortic root replacement with concomitant aortic valve replacement (Bentall procedure) instead of VSARR (David-I procedure) for these patients.

### Limitations

The study is a retrospective, single-center study. The follow-up echocardiographic data were exclusively gathered from referring cardiologists. Furthermore, the BAV cohort was relatively small.

## Conclusions

The aortic valve–sparing David-I procedure in patients with BAV morphology demonstrates excellent short-term results including aortic valve competency and good long-term outcomes regarding valve-related complications such as stroke and endocarditis. However, we observed a significantly higher rate of severe AR during follow-up, leading to a greater need for redo procedures. The perioperative risk analysis, particularly concerning severe complications such as hemorrhage, stroke, and low cardiac output syndrome, indicates that the David procedure is a safe and effective technique when performed electively. Patient selection and low threshold to convert to a Bentall procedure are key to ensure not only safety and excellent short-term outcomes but also satisfactory long-term outcomes in patients with aortic root aneurysms. In patients with high-grade aortic insufficiency, a Bentall procedure should be considered as the primary surgical strategy.

## Conflict of Interest Statement

M.K. is a physician proctor and a member of the medical advisory board for Sanamedi, is a physician proctor for Peter Duschek, is a medical consultant for EVOTEC and Moderna, and has received speakers' honoraria from EDWARDS, AtriCure, Medtronic, and Terumo. All other authors reported no conflicts of interest.

The *Journal* policy requires editors and reviewers to disclose conflicts of interest and to decline handling or reviewing manuscripts for which they may have a conflict of interest. The editors and reviewers of this article have no conflicts of interest.
